# Seroprevalence of *Toxoplasma gondii* Infection in Veterinary Medicine Professionals and Students in Aguascalientes, Mexico

**DOI:** 10.3390/epidemiologia7030061

**Published:** 2026-05-04

**Authors:** Isabel de Velasco-Reyes, Saúl Emmanuel Torres-García, José de Jesús Hernández-Rangel, Adriana Cruz-Bañares, Juan Luis Chávez-Chávez, Carlos Cruz-Vázquez

**Affiliations:** 1 Tecnológico Nacional de México, Instituto Tecnológico El Llano Aguascalientes, Km 18 Carretera Aguascalientes a San Luis Potosí, El Llano 20330, Aguascalientes, Mexico; cruva18@yahoo.com.mx; 2 Campus Aguascalientes, Universidad Cuauhtémoc, Av. Adolfo López Mateos No. 103-2, Aguascalientes 20908, Aguascalientes, Mexico; 3219@ucuauhtemoc.edu.mx (S.E.T.-G.); 5140@ucuauhtemoc.edu.mx (J.L.C.-C.); 3 Centro de Ciencias Agropecuarias, Universidad Autónoma de Aguascalientes, Av. Universidad No. 940, Aguascalientes 20100, Aguascalientes, Mexico; jesus.hernandezr@edu.uaa.mx; 4 Facultad de Medicina, Universidad Autónoma de Yucatán, Av. Itazes No. 498, Merida 97000, Yucatan, Mexico; adriana.cruz@correo.uady.mx

**Keywords:** Mexico, seroprevalence, *Toxoplasma gondii*, veterinary students, veterinarians

## Abstract

Background/Objectives: Toxoplasmosis is a globally distributed parasitic zoonosis caused by *Toxoplasma gondii* (Apicomplexa, Sarcocystidae), an obligate intracellular protozoan with an indirect life cycle in which domestic cats and wild felids serve as definitive hosts, whereas humans and a broad range of domestic and wild animals act as intermediate hosts. The objective of the study was to document the seroprevalence of anti-*Toxoplasma gondii* antibodies in professionals and students of Veterinary Medicine in Aguascalientes, Mexico. Methods: The study included 153 clinically healthy individuals from two population segments: Veterinarians (70) and Veterinary Medicine Students (83). Serum samples were analyzed using a commercial ELISA test to determine the presence of *T. gondii*-specific IgG. A questionnaire was applied to collect sociodemographic information and information about contact with cats. Results: The overall prevalence of anti-*T. gondii* antibodies in the study population was 7.8% (12/153; CI 95% 4.3–13.6). In the group of Veterinarians, the seroprevalence was 11.4% (8/70; CI 95% 5.4–21.8), while in the group of students it was 4.8% (4/83; CI 95% 1.5–12.5), with no differences observed between them (*p* = 0.22). Association was found with those who consume raw/undercooked meat (*p* = 0.002). Conclusions: In this cross-sectional sample of veterinary professionals and students in Aguascalientes, anti-*T. gondii* IgG seroprevalence was 7.8%, with no statistically significant difference between occupational groups. Consumption of raw or undercooked meat was the only exposure significantly associated with seropositivity.

## 1. Introduction

Toxoplasmosis is a globally distributed parasitic zoonosis caused by *Toxoplasma gondii* (Apicomplexa, Sarcocystidae), an obligate intracellular protozoan with an indirect life cycle in which domestic cats and wild felids serve as definitive hosts, whereas humans and a broad range of domestic and wild animals act as intermediate hosts [1].

Toxoplasmosis represents a significant public health problem. It has been estimated that the seroprevalence worldwide ranges from 30 to 35%, with significant variations by region, and that at least one third of the population has been exposed to the parasite. However, clinical manifestations are infrequent, so most infections are considered asymptomatic, although some people may experience symptoms ranging from mild to severe [2,3]; recently, a literature review identified a seroprevalence of 41% in people exposed to the parasite for work-related reasons, and 27% in veterinarians in particular [4].

In Mexico, seroprevalence in the general population has been found to be 27.3%, with variations between regions and risk group [5,6]. People can contract the infection through various routes, primarily by consuming viable cysts of the parasite present in raw or undercooked meat and by ingesting sporulated oocysts excreted in the feces of infected cats, which are present in the environment and can contaminate water and food; close contact with cats has been documented to be highly associated with infection [1,7,8].

Veterinary professionals, as well as students in training, interact daily with small and large animals, as these are essential to their work in preserving animal health. Consequently, they are continuously exposed to *T. gondii* infection, regardless of feeding habits, which could be another source of infection. However, the epidemiology of *T. gondii* infection in these population groups has been scarcely studied [4], particularly in Mexico [9].

The objective of the study was to document the seroprevalence of anti-*T. gondii* antibodies in professionals and students of Veterinary Medicine in Aguascalientes, Mexico.

## 2. Materials and Methods

### 2.1. Study Site

This study was carried out in the state of Aguascalientes, Mexico, located in the north-central region of the country (21°52′54″ N, 102°17′28″ W) at an altitude between 1860 and 2010 m above sea level, with an average annual temperature of 18 °C, with a semi-dry weather and rainy regime in the summer.

### 2.2. Study Population and Samples

A cross-sectional study was conducted between May and September 2025. For this purpose, sampling was carried out using the non-probabilistic convenience method as described below. Private small animal clinics, animal production units, and veterinary schools were visited. Veterinarians and students were invited to participate in the study. Inclusion criteria included at least one year of experience handling animals and being over 18 years of age, regardless of gender. All participants read and signed the informed consent form, which described the purpose and procedures of the study.

The study included 153 clinically healthy individuals from two population segments: Veterinarians (70) and Veterinary Medicine Students (83). A blood sample was taken by puncture of the median cubital vein using a new Vacutainer set. The serum was obtained by centrifugation at 1000 g for 10 min and placed in polystyrene vials for storage at −20 °C until use in the serological test.

The investigation was conducted in accordance with the Declaration of Helsinki, and the protocol was approved by the Research Ethics Committee of Instituto Tecnológico El Llano Aguascalientes (ITEL-CEI/003/2025 on 31 March 2025).

### 2.3. Serological Test

Serological testing for *T. gondii* exposure was performed using a commercial enzyme-linked immunosorbent assay (ELISA) for the qualitative detection of anti-*T. gondii* IgG antibodies in serum (Bio-Toxo IgG, Grupo Mexlab, Mexico City (CDMX), Mexico). All procedures were conducted in strict accordance with the manufacturer’s instructions. Serum samples, together with positive and negative controls, were diluted 1:21 and analyzed in duplicate. Absorbance was measured at 450 nm, and an antibody index was calculated as the ratio of the optical density of each sample to the assay cut- off value. Results were interpreted as follows: an antibody index < 0.90 was considered negative, 0.90–1.10 was considered doubtful, and >1.10 was considered positive. According to the manufacturer, the assay demonstrates 95% concordance with a reference ELISA method.

### 2.4. Questionnaire

A closed questionnaire was applied to each participant in order to record some sociodemographic attributes such as age, gender, eating and hygiene habits as well as information about their professional practice in relation to possible contact with cats or responsible ownership of them (the complete questionnaire is available as Supplementary Materials File S1 Questionnaire). The attributes considered for further analysis were ordered and categorized as shown in tables.

### 2.5. Data Analysis

The general prevalence of anti-*T. gondii* antibodies were calculated in each of the population groups and according to the different attributes identified in the questionnaires with their respective Confidence Interval (CI). The seroprevalence observed in the population segments was examined with Chi-square test with Yates correction at *p* < 0.05. All procedures were performed using the Epi Info 3.5.1 software package.

## 3. Results

The overall prevalence of anti-*T. gondii* antibodies in the study population was 7.8% (12/153; CI 95% 4.3–13.6). In the group of veterinarians, the seroprevalence was 11.4% (8/70; CI 95% 5.4–21.8), while in the student group it was 4.8% (4/83; CI 95% 1.5–12.5), with no significant differences observed between the two groups (*p* = 0.22).

Table 1 presents seroprevalence estimates for the combined study population according to the variables evaluated; only one variable showed a statistically significant association with seropositivity. This was among people who reported that they usually consume raw or undercooked meat; this group had a higher seroprevalence than those who do not usually eat meat in this way (*p* < 0.002). Despite the seroprevalence was higher in males and in the 45–54 age group, however, no statistically significant association was identified.

Table 2 shows the distribution of seroprevalence in the group of veterinarians according to the different attributes recorded in the survey. In none of the categories of the different variables studied were statistical differences observed; however, it can be observed that seroprevalence was higher in males and that veterinarians aged 25 to 34 years had lower seroprevalence than older age groups.

The seroprevalence for the student group, according to the different attributes recorded in the survey, is shown in Table 3. In none of the categories of the different variables studied were statistical differences observed; however, it can be mentioned that the seroprevalence was similar between male and female, and that in the 25–34 age group, a higher seroprevalence was identified.

## 4. Discussion

Toxoplasmosis is a parasitic zoonosis considered an occupational risk disease for people dedicated to animal health care due to their daily contact with various domestic and wild species [4]. In the present study, a general seroprevalence of 7.8% was identified in the study population, higher in Veterinarians (11.4%), than in Veterinary Medicine students (4.8%). At the start of our study, we expected the seroprevalence in the population under scrutiny to be moderately high, since the prevalence of antibodies in domestic animals reported by our working group in the state of Aguascalientes had been consistently high, particularly in species that can be considered indicators of environmental contamination by oocysts of the parasite, such as backyard chickens (67%) and dogs (59%). In the latter case, the seroprevalence in rural dogs was 74%, in stray dogs 60%, and in pets 32% [10,11], this could mean the existence of an environment contaminated with oocysts of the parasite, excreted by cats, which would be the main source of infection to intermediate hosts through the consumption of contaminated water and food, as has been suggested in the literature, since the cat population in rural and urban areas has a high density, wide distribution and unquestionable importance throughout the world, it is estimated that at least 2.6% of cats excrete oocysts [8,12], which, due to their biological characteristics, widely pollute the environment [13]. However, there appears to be no direct relationship between the frequencies of anti-*T. gondii* antibodies in the aforementioned domestic animals and that identified in the studied human population.

In a case–control study conducted in the city of Durango, Mexico, a seroprevalence of 6% has been reported in people exposed to animals, with no difference from that identified in the non-exposed group (5.5%) and among veterinarians (7.2%) [14]; in another study conducted among small animal veterinarians and pet groomers in two cities in the state of Veracruz, Mexico, the seroprevalence was 2.2%, with no positive cases identified in the groomer group [9]. Interestingly, similar studies in veterinarians have reported a seroprevalence of 8% in Korea [15], 8.7% in India [16], and 14.2% in Canada [17], while in veterinary medicine students in Brazil, 16.1% of cases were identified as seropositive [18], and in the United States of America, 5.6% was found [19]. Our results are consistent with the cited studies, confirming that, at least according to these works, exposure to animals does not necessarily represent a high risk of infection. It seems clear that domestic animals are more exposed to contact with *T. gondii* oocysts, probably due to factors related to management and hygiene practices in the places where they live and develop.

Due to the small number of positive cases identified in the present study, it was not possible to identify differences between the attributes of the general population or in the two groups that made it up, nor could the seroprevalence be associated with any of the attributes to identify risk factors for infection. However, the presence of positive cases can provide information for understanding the dynamics of the infection. One factor to consider in this endeavor is the contact that animal health professionals have with cats through their clinical care and/or cat ownership, where a high number of positive cases were found. However, the presence of positive cases can provide information for understanding the dynamics of the infection. It should be noted that infection does not necessarily occur through physical contact, as foodborne transmission—whether through the consumption of water or food contaminated with oocysts excreted by cats with an active infection, or through the consumption of raw or undercooked meat containing the parasite’s cysts—is a route of recognized epidemiological importance. This latter situation was present in the majority of positive cases. These routes of infection are important and have been widely recognized and documented in the literature [1,8,13].

Other population attributes such as gender and age can also be considered indicators; however, due to the moderate number of respondents and the fact that no statistically significant association was identified, the information should be interpreted with caution. In the present study, more seropositive cases were observed in males. This trend has been recognized in other studies, and globally, it has been observed that males who work with animals are up to 1.7 times more likely to be infected than females [4]. Regarding age, most cases were observed in the 35–44 age range, which is considered a stage of extensive professional activity and therefore probably greater exposure. In another study conducted in Mexico, the 20–44 age range was found to have the highest prevalence among small animal veterinarians [9].

The present study had some limitations, the most relevant being the sample size and the small number of positive cases identified, which did not allow for the development of a risk analysis that would provide elements to document the epidemiology of the infection in the groups under scrutiny.

## 5. Conclusions

In conclusion, in this cross-sectional sample of veterinary professionals and students in Aguascalientes, anti-*T. gondii* IgG seroprevalence was 7.8%, with no statistically significant difference between occupational groups. Consumption of raw or undercooked meat was the only exposure significantly associated with seropositivity.

## Figures and Tables

**Table 1:** Prevalence of anti-*T. gondii* antibodies in the study population according to different attributes.

Attribute		Samples	Positives	Prevalence (%)	95% CI ^1^	*p* Value
Gender						0.26
	Female	93	5	5.3	2–12.6	
	Male	60	7	11.6	5.2–23.1	
Age (years)						0.95
	19–24	63	3	4.7	1.2–14.1	
	25–34	44	3	6.8	1.7–19.7	
	35–44	29	3	10.3	2.7–28.4	
	45–54	10	2	20	3.5–55.7	
	>55	7	1	14.2	0.75–58	
Contact with cats						0.79
	Yes	115	10	8.7	4.4–15.8	
	No	38	2	5.2	0.92–19	
Type of contact						0.96
	Clinical care	62	6	9.6	4–20.5	
	Pet ownership	53	4	7.5	2.4–19	
	No contact	38	2	5.2	0.92–19	
Contact with cat feces						0.82
	Yes	100	7	7	3.1–14.3	
	No	53	5	9.4	3.5–21.4	
Wash vegetables						0.84
	Yes	131	11	8.4	4.4–14.8	
	Sometimes	22	1	4.5	0.24–24.8	
Eats undercooked/raw meat						0.002
	Yes	40	8	20	9.6–36.1	
	No	113	4	3.5	1.1–9.3	
Where they eat						0.94
	Street stall	14	1	7.1	0.37–35.8	
	Restaurant	15	2	13.3	2.3–41.6	
	Home	124	9	7.2	3.5–13.7	

^1^ CI: Confidence Interval.

**Table 2:** Prevalence of anti-*T. gondii* antibodies in Veterinarians according to different attributes.

Attribute		Samples	Positives	Prevalence (%)	95% CI ^1^	*p* Value
Gender						0.13
	Female	39	2	5.1	0.89–18.6	
	Male	31	6	19.3	8.1–38	
Age (years)						0.49
	25–34	32	2	6.2	1–22.2	
	35–44	21	3	14.2	3.7–37.3	
	45–54	10	2	20	3.5–55.7	
	>55	7	1	14.2	0.75–58	
Contact with cats						0.98
	Yes	57	6	10.5	4.3–22.2	
	No	13	2	15.3	2.7–46.3	
Type of contact						0.87
	Clinical care	43	4	9.3	3–23	
	Pet ownership	14	2	14.2	2.5–43.8	
	No contact	13	2	15.3	2.7–46.3	
Contact with cat feces						0.81
	Yes	42	4	9.5	3–23.5	
	No	28	4	14.2	4.6–33.5	
Wash vegetables						0.92
	Yes	66	7	10.6	4.7–21.2	
	Sometimes	4	1	25.0	1.3–78	
Eats undercooked/raw meat						0.36
	Yes	21	4	19	6.2–42.5	
	No	49	4	8.1	2.6–20.4	
Where they eat						0.61
	Street stall	9	0	0	-	
	Restaurant	8	2	25.0	4.4–64.4	
	Home	53	6	11.3	4.6–23.7	

^1^ CI: Confidence Interval.

**Table 3:** Prevalence of anti-*T. gondii* antibodies in Veterinary Medicine students according to different attributes.

Attribute		Samples	Positives	Prevalence (%)	95% CI ^1^	*p* Value
Gender						0.91
	Female	54	3	5.5	1.4–16.3	
	Male	29	1	3.4	0.18–19.6	
Age (years)						0.28
	19–24	63	2	3.1	0.55–11.9	
	25–28	12	2	16.6	2.9–49.1	
	>29	8	0	0	-	
Contact with cats						0.43
	Yes	58	4	6.9	2.2–17.5	
	No	25	0	0	-	
Type of contact						0.85
	Clinical care	19	2	10.5	1.8–34.5	
	Pet ownership	38	2	5.2	0.92–19	
	No contact	25	0	0	-	
Contact with cat feces						0.74
	Yes	58	3	5.1	1.3–15.3	
	No	25	1	4	0.21–22.3	
Wash vegetables						0.64
	Yes	65	4	6.1	1.9–15.7	
	Sometimes	18	0	0	-	
Eats undercooked/raw meat						0.61
	Yes	19	1	5.2	0.27–28.1	
	No	64	3	4.6	1.2–13.9	
Where they eat						0.85
	Street stall	5	1	20	1–70.1	
	Restaurant	7	0	0	-	
	Home	71	3	4.2	1.1–12.6	

^1^ CI: Confidence Interval.

## Data Availability

The data presented in this study are available on request from the corresponding author due to privacy reasons.

## Supplementary Materials

The following supporting information can be downloaded at: https://www.mdpi.com/article/10.3390/epidemiologia7030061/s1, File S1: Questionnaire.
