# Supplementary material for: Seroprevalence of Toxoplasma gondii Infection in Veterinary Medicine Professionals and Students in Aguascalientes, Mexico
Source: Epidemiologia (Basel). 2026 May 4;7(3):61. doi: 10.3390/epidemiologia7030061 (PMC13214915; doi:10.3390/epidemiologia7030061)
Supplement: Supplementary file 1 [file epidemiologia-07-00061-s001.zip › epidemiologia-4189542-supplementary.pdf]

TECNOLÓGICO NACIONAL DE MÉXICO®

ITEL
INSTITUTO TECNOLÓGICO EL LLANO AGUASCALIENTES

QUESTIONNAIRE

ID __________

Name ____

Age ________ years phone number ______________________

Sex: ☐ female ☐ male

Occupation: ☐ student ☐ veterinarian

Hygiene habits

Do you wash your hands before eating? ☐ yes ☐ no

Do you wash your hands after using the restroom? ☐ yes ☐ no

Do you wash your hands before cooking food? ☐ yes ☐ no

Eating habits

Do you wash the fruits and vegetables you consume? ☐ yes ☐ no

If the answer is yes, how do you wash them? ☐ tap water ☐ bottled water ☐ boiled or chlorinated water ☐ soap and chlorine

Do you consume meat that is undercooked or raw? ☐ yes ☐ no

Most of the time, where do you eat your meals at: ☐ home ☐ restaurant ☐ street stall

Contact with cats

Do you have direct contact with cats? ☐ yes ☐ no

☐ Clinical care ☐ Pet ownership ☐ No contact _____________

Do you have contact with cat feces? ☐ yes ☐ no

How are the cat feces handled? ☐ with gloves ☐ litter scoop ☐ broom and dustpan

How do you dispose of the cat feces? ☐ in a container ☐ buried ☐ into the environment

Have you undergone serological tests for the detection of toxoplasmosis?

☐ yes ☐ no

Result: ☐ positive ☐ negative
